# Helminths and protozoan parasites in common opossums (*Didelphis marsupialis*) in a suburban area in Medellín, Colombia

**DOI:** 10.1590/S1984-29612024082

**Published:** 2025-02-14

**Authors:** Luisa Arango López, Daisy Alejandra Gómez-Ruiz, Gloria Yaneth Sánchez-Zapata, Laura Marcela Gutiérrez-Giraldo, Natalia María Granda-Orozco, Cristina Úsuga-Monroy, Horwald Alexander Bedoya Llano

**Affiliations:** 1 Grupo de Investigación GINVER, Corporación Universitaria Remington, Facultad de Medicina Veterinaria, Medellín, Colombia

**Keywords:** Endoparasites, helminth fauna, marsupials, nematodes, zoonosis, Endoparasitas, helmintofauna, marsupiais, nematodes, zoonoses

## Abstract

*Didelphis marsupialis* is a marsupial species that effectively adapts to synanthropic processes developing in cities. This marsupial lives closely with domestic animals and humans, which has favored the active exchange of parasites, thus increasing polyparasitism. Hence, this study aimed to determine the prevalence of helminths and protozoans infecting *D. marsupialis* in the Corregimiento of Santa Elena, Medellín. Twenty-three individuals were captured and classified as male, female, adult, or juvenile. The fecal samples were analyzed using various coprodiagnostic techniques. The eggs and oocysts were identified by microscopic evaluation of their morphology and morphometry. Twelve parasite species were identified: nine nematodes, one acanthocephalan, and two protozoans. In addition, an Adeleid coccidia considered pseudoparasite was found. The most prevalent parasite species (>50%) were *Eimeria* sp, *Cruzia* sp., *Aspidodera* sp., and *Gnathostoma turgidum*, and nematode larvae. No significant differences were observed between parasite prevalence and host sex or age. Parasites of public health interest, such as *Trichuris* spp., Capillariidae nematodes, *Strongyloides* spp., and *Giardia* spp., were also identified. This study confirmed that the urban habitat of the opossum has a high frequency and diversity of endoparasites, some of which have been reported for the first time in Colombia.

## Resumo

*Didelphis marsupialis* é uma espécie que se adapta aos processos sinantrópicos que vêm desenvolvendo-se nas cidades. Esse marsupial convive com animais domésticos e humanos, o que tem favorecido a troca ativa de parasitas, que aumenta o poliparasitismo. Assim, este estudo tem como objetivo determinar a prevalência de helmintos e protozoários que infectam *D. marsupialis* na localidade de Santa Elena, Medellín. Vinte e três indivíduos foram capturados e classificados como macho, fêmea, adulto ou juvenil. As amostras fecais foram analisadas usando técnicas de coprodiagnóstico. Os ovos e os oocistos foram identificados por avaliação microscópica de sua morfologia e morfometria. Foram identificadas 12 espécies de parasitos: nove nematodes, um acantocéfalo e dois protozoários. Além disso, foi encontrado um coccídio Adeleid, considerado pseudoparasita. As espécies de parasitos mais prevalentes (>50%) foram *Eimeria* sp, *Cruzia* sp., *Aspidodera* sp. e *Gnathostoma turgidum*, e larvas de nematodes. Não foram observadas diferenças significativas na prevalência entre sexo e idade. Também foram identificados parasitas de interesse para a saúde pública, como *Trichuris* spp., nematódeos Capillariidae, *Strongyloides* spp. e *Giardia* spp. Este estudo confirmou que o habitat urbano apresenta uma alta frequência e diversidade de endoparasita nos gambás aqui estudados. Alguns desses achados são relatados pela primeira vez na Colômbia.

## Introduction

The common opossum (*Didelphis marsupialis*) is a terrestrial mammal widely distributed throughout the Americas, from Mexico to northeastern Argentina (Caramaschi et al., 2013). These animals inhabit forest, areas with dense vegetation and peridomestic spaces, exhibit nocturnal habits, and possess an omnivorous diet that ranges from small invertebrates to fruits and seeds (Flórez-Oliveros & Vivas-Serna, 2020). Due to these characteristics, the common opossum is recognized as a species with seed dispersal potential that contributes to the biological control of small vertebrates and invertebrates (Cantor et al., 2010; Ramírez & Osorio, 2014).

Parasites play a crucial role in wildlife because their interaction with hosts, the environment influence the emergence of zoonotic diseases and the complex dynamics of transmission, highlighting the need to understand and address these interactions in a One Health context (Rizzoli et al., 2019). Due to its remarkable ability to adapt to various environments and resources found in human-modified landscapes, the common opossum is considered a synanthropic species that serves as an intermediate or final host for a variety of endo-and ectoparasite species (Teodoro et al., 2019). This species is of considerable significance in the life cycles of protozoans, helminths, and arthropods, some of which are important pathogens or disease vectors for humans and other animals (Bezerra-Santos et al., 2021). However, gaps remain in our understanding of these interactions, particularly regarding endoparasitic relationships.

Several studies in the Americas have documented the presence of gastrointestinal helminths and protozoan species in *D. marsupialis*. However, these studies reported different taxonomic resolutions depending on the data source, such as feces or dead animals. There are reports of 13 nematode species, nine trematodes species, two cestode species, three acanthocephalan species, and coccidias in *D. marsupialis* (Chagas-Moutinho et al., 2007; Acosta-Virgen et al., 2015; López-Caballero et al., 2015; Valerio-Campos et al., 2015; Chero et al., 2017; Polo-Gonzales et al., 2019; Freitas et al., 2022). Additionally, among the zoonotic species associated with opossums, worldwide reports have highlighted the presence of the helminths *Paragonimus caliensis*, and *Paragominus mexicanus* (Bezerra-Santos et al., 2021).

Studies regarding parasites of *D. marsupialis* in Colombia are limited. Ramírez & Osorio (2014) analyzed 15 feces samples and 10 gastrointestinal content samples from *D. marsupialis* (n = 15) and revealed the presence of adults and eggs of the following helminths: *Ancylostoma* sp., *Dipylidium* sp., *Macracanthorhynchus* sp., *Physaloptera* sp., *Strongyloides* sp., and *Trichuris* sp. Moreover, in a coproparasitological analysis of *D. marsupialis* (n = 4), Muñoz Rodríguez et al. (2017) reported the presence of *Ancylostoma* sp. and *Trichuris* sp. helminths and protozoans belonging to the genus *Eimeria*. Betancourt-Echeverri et al. (2021) reported the presence of adult nematodes in the gastrointestinal tract of *D. marsupialis* (n = 2) belonging to the genera *Aspidodera*, *Capillaria*, *Cruzia*, *Physaloptera*, *Travassostrongylus*, and *Trichuris*. Additionally, in the same study, eggs of the superfamily Ascaroidea, Spiruroidea, and Trichuroidea were observed in the feces. Some of these parasites are of public health importance and have the risk of possible transmission to humans. These findings highlight the need for further research to expand our understanding of parasitic fauna associated with *D. marsupialis* in Colombia.

Additional studies are needed to fill the knowledge gaps in this area and provide valuable insights into the diversity and impact of parasites on the common opossum population. Furthermore, given that the viable population of *D. marsupialis* in urban and peri-urban ecosystems meets that of humans and domestic animals (Saldaña Garro et al., 2019), understanding the parasites in this context is crucial. Therefore, the objective of this study was to report the richness and prevalence of endoparasites from *D. marsupialis* in a peri-urban area located in the Cordillera Central region of Colombia.

## Material and Methods

### Study area

**Figure 1:**
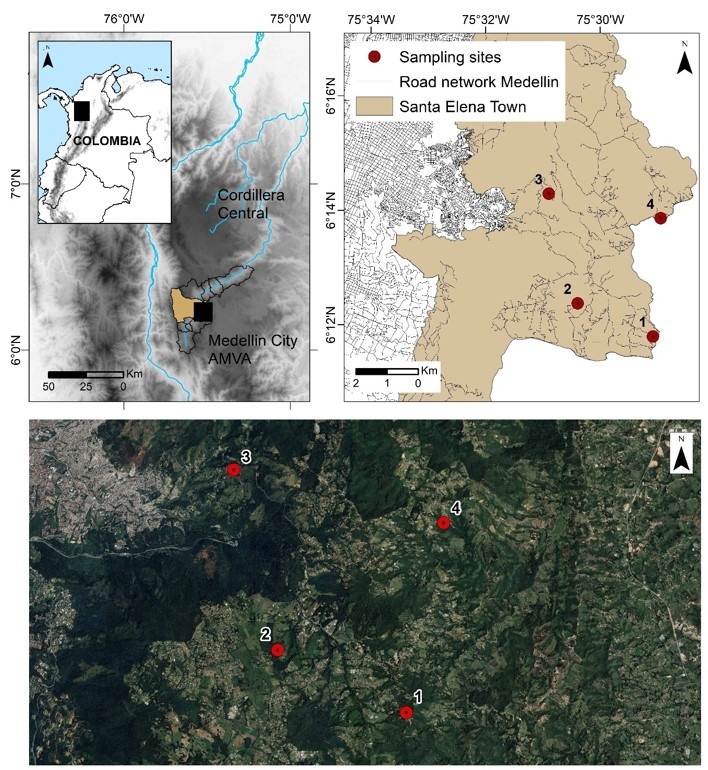
Maps showing study sampling sites in the Corregimiento of Santa Elena, Medellín, Colombia. (1) Cerro Verde; (2) Paysandú; (3) Clínica Veterinaria Uniremington; (4) Barro Blanco.

This study was conducted at four sites located in the Corregimiento region of Santa Elena, Medellín, Colombia. Santa Elena is characterized by an average temperature of 17 °C and an altitude of 2,573 m above sea level, with an economic activity based on crops, flowers, and tourism. Based on the vegetation cover and species movement friction maps (Colorado Zuluaga et al., 2017), four localities were selected for the opossum capture: Cerro Verde (6°19'93.35”N, −75°48'50.12”W), Paysandú (6°20'60.47”N, −75°01'98.03”W), Clínica Veterinaria Uniremington (CVU; 6°23'85.91”N, −75°51'45.76”W), and Barro Blanco (6°24'40.43”N, −75°48'20.50”W) (Figure 1). The study area comprised small fragments of native vegetation within an urbanized environment.

### Capture of opossums

Between June to December 2022, eight field trips were carried out, in which 10 Tomahawk®-type live capture traps (66 × 23 × 23 cm) were placed 10 m from each other along corridors of dense vegetation, preferably bordering beds, streams, crops and/or peridomiciliary areas. Captures were conducted for three nights at each study site. This was equivalent to 80 traps per study site each night. The traps were baited with sardines or bananas, checked every morning, and rebaited if necessary. Sex, weight, and age data were also recorded. Body size was used as a measure of host age (Arcangeli, 2014). After samples collection, all the individuals were released within the shortest possible time to reduce stress.

This study was approved by the Bioethics Committee for Animal Research of the Medicine Veterinary Faculty, Corporación Universitaria Remington (CIBA-Acta No. 03–2021).

### Sample collection

Fecal samples were collected directly from the animal or the trap and stored in 50-mL Falcon tubes preserved in 70% alcohol. Tubes were transported at room temperature to the CVU parasitology laboratory a few hours after collection. Upon arrival, the samples were analyzed using the direct coproparasitology method (Kaminsky, 2014). When a sufficient fecal sample was available, the Baermann method was used to recover nematode larvae from the fecal samples (Kaminsky, 2014). All samples were refrigerated at 4 °C, and subsequently processed and analyzed individually by two qualitative coproparasitological methods: centrifugation-flotation in sucrose solution (d = 1,203 g/cm^3^) (Sheather) and centrifugation-sedimentation in formalin-ether (Ritchie) (Pena et al., 1997). Additionally, samples positive for coccidia were sporulated with dichromate potassium solution (K_2_Cr_2_O_7_) at 2.5% for five days (Lassen & Seppä-Lassila, 2014). Morphological studies of parasite eggs and oocysts/cysts were conducted using optical microscopy to determine the shape and size of these structures and to compare them with the taxonomic keys used in other studies (Rodríguez Vivas & Galera, 2005; Duszynski, 2016; Teodoro et al., 2019; Bezerra-Santos et al., 2020; Boullosa et al., 2021).

### Data analysis

Based on Bush et al. (1997), the prevalence of each parasite species was calculated using the following formula: No. of positive animals / total population × 100 using confidence interval of 95%. The chi-square test was used to determine the association between the prevalence of parasite infection and two categorical variables: sex and age. Statistical analyses were performed using R software for Windows, version 3.2.1 (R Core Team, 2022).

## Results

In total, 23 *D. marsupialis* individuals were captured and only one recapture was recorded during the sampling period. Of these individuals, 15 (65.2%) were male, six (26.1%) were female, 15 were adults (65.2%), and six were juveniles (26.1%). It was not possible to determine the sex and age of two individuals as they fled at the time of manipulation *in situ.* Regarding capture locations, 11 (47.8%) were captured in Barro Blanco, 9 (39.1%) in the CVU, two (8.7%) in Cerro Verde, and one (4.3%) in Paysandú.

All analyzed samples contained one or more parasitic forms (eggs/oocysts). Helminth eggs were detected in 100.0% (23/23) of the samples, whereas protozoan oocysts were observed in 86.95% (20/23). Helminth parasites were categorized as nine nematodes and one acanthocephalan. Two protozoan species belonging to the families Hexamitidae and Eimeriidae were also identified. In addition, an Adeleid coccidia considered as a pseudoparasite was found.

**Figure 2:**
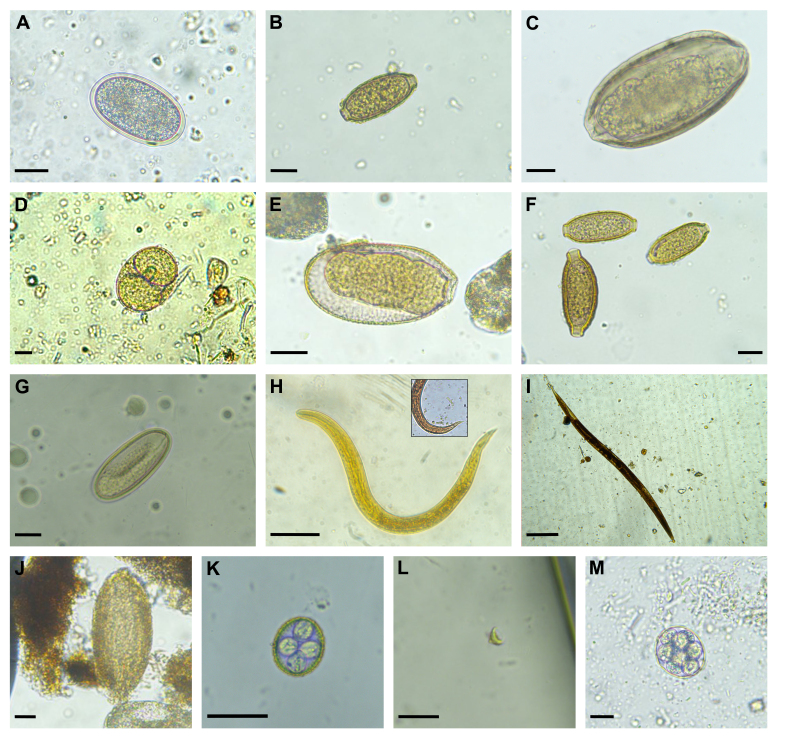
Helminth eggs and protozoan cyst/oocysts recovered from faecal samples of *Didelphis marsupialis* found in the Corregimiento of Santa Elena, Medellín, Colombia. (a) *Aspidodera* sp. Egg; (b) Capillariidae egg; (c) *Cruzia* sp. Egg; (d) *Strongyloides* sp. egg (note the larva emerging from the egg); (e) *Gnathostoma turgidum* egg; (f) Trichuridae eggs; (g) *Turgida* sp. Egg; (h) Metastrongyloidea larvae; (i) Nematode larvae; (j) *Oligacanthorhynchus microcephalus* egg; (k) Coccidian *Eimeria* sp. sporulated oocyst; (l) *Giardia* sp. Cyst; (m) *Octosporella* sp. sporulated oocyst. Scale bar = 20 um.

The nematode eggs were identified as: *Aspidodera* sp. (Heterakoidea; Aspidoderidae) (Figure 2a), Capillariidae nematodes (Trichinelida: Capillariidae) (Figure 2b), *Cruzia* sp. (Cosmocercoidea; Kathlaniidae) (Figure 2c), *Gnathostoma turgidum* (Rhabditida: Gnathostomatidae) (Figure 2d), *Strongyloides* sp. (Rhabditida: Strongyloidoidea) (Figure 1e), Trichuridae eggs (Trichinellida: Trichuridae) (Figure 2f), and *Turgida* sp. (Spiruroidea; Physalopteridea) (Figure 2g). We also detected a pulmonary species classified at the superfamily level as Metastrongyloidea (Strongylida: Angiostrongylidae) (Figure 2h) and an unclassified larval species (Figure 2i). The acanthocephalan species *Oligacanthorhynchus microcephalus* (Oligacanthorhynchida: Oligacanthorhynchidae) (Figure 2j) was identified. In terms of protozoans, *Eimeria* sp. oocysts (Coccidia: Eimeridae) (Figure 2k) and *Giardia* sp. cysts (Diplomonadida: Hexamitidae) (Figure 2l) were observed. Finally, we reported the genus *Octosporella* (Pezizales: Pyronemataceae) as a spurious parasite (Figure 2m). The morphometric characteristics of the parasites are listed in Table 1.

**Table 1:** Average measurement of helminth eggs and protozoan cysts/oocysts recovered from *Didelphis marsupialis* fecal samples in the Corregimiento of Santa Elena, Medellín, Colombia. Length (µm), width (µm), and their standard deviations (± SD).

**Parasite**	**Number of eggs measured**	**Length**	**Width**
Nematodes			
*Aspidodera* sp.	19	68.1 ± 2.0	39.7 ± 2.2
*Capillaria-*like type	14	56.8 ± 2.8	25.5 ± 1.9
*Cruzia* sp.	23	109.0 ± 11.5	56.2 ± 6.6
*Gnathostoma turgidum*	10	97.8 ± 1.9	41.7 ± 2.1
Metastrongyloidea larvae	7	218.4 ± 24.8	-
Nematode larvae	18	238.2 ± 55.7	-
*Turgida* sp.	4	7414.3 ± 2.1	32.5 ± 3.3
*Strongyloides* sp.	2	45.9 ± 22.1	28.2 ± 7.9
*Trichuris-*like type	7	66.8 ± 3.5	28.5 ± 2.9
*Oligacanthorhynchus microcephalus*	1	91.3	42.0
Protozoans			
*Eimeria* sp.	35	22.3 ± 3.4	-
*Giardia* sp.	2	9.6 ± 1.4	-
*Octosporella* sp.	10	44.3 ± 6.9	36.8 ± 3.4

- Data not available.

**Figure 3:**
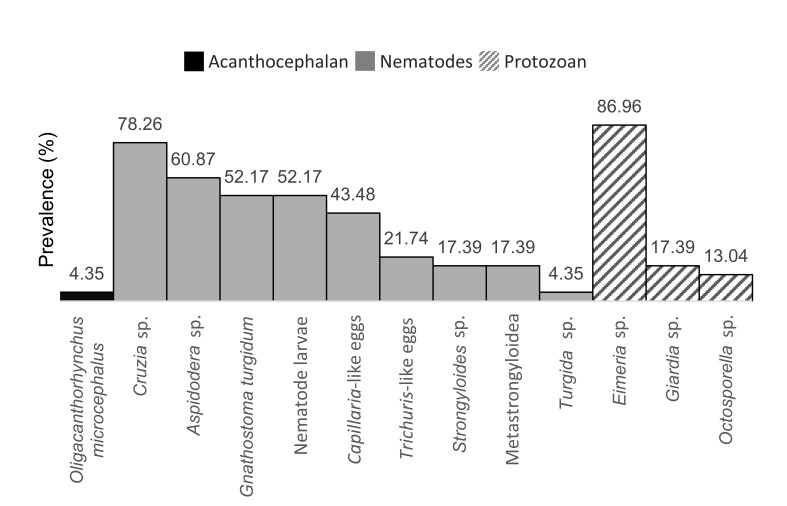
Prevalence of helminths and protozoan found in *Didelphis marsupialis* from the Corregimiento of Santa Elena, Medellín, Colombia.

The data presented in Figure 3 show that the highest prevalence values were observed for the protozoan *Eimeria* sp., followed by the nematode *Cruzia* sp. No significant relationship was observed between parasite prevalence and host sex or age (Table 2).

**Table 2:** Chi-square and p-values for prevalence of helminth and protozoan parasites found in *Didelphis marsupialis* in the Corregimiento of Santa Elena, Medellín, Colombia in relation to host sex and age (N = 23; N_male_ = 15; N_female_ = 6; N_adult_ = 15; N_juvenile_ = 6).

**Parameters**	***Aspidodera* sp.**		***Capillaria-*like eggs**		***Cruzia* sp.**		***Gnasthostoma turgidum***		**Metastrongyloidea larvae**		**Nematode larvae**		***Strongyloides* sp.**		***Turgida* sp.**		***Trichuris*-like eggs**		***Oligacanthorhynchus microcephalus***		***Eimeria* sp.**		***Giardia* sp.**		***Octosporella* sp.**		
	***X^2^***	***p***	***X^2^***	***p***	***X^2^***	***p***	***X^2^***	***p***	***X^2^***	***p***	***X^2^***	***p***	***X^2^***	***p***	***X^2^***	***p***	***X^2^***	***p***	***X^2^***	***p***	***X^2^***	***p***	***X^2^***	***p***	***X^2^***	***p***
**Sex**	0.311	0.577	0.019	0.890	2.625	0.105	0.019	0.890	0.038	0.843	0.687	0.407	2.626	0.105	1.111	0.291	2.625	0.105	0.884	0.347	0.42	0.516	0.497	0.480	1.111	0.291
**Age**	0.311	0.577	0.019	0.890	0.236	0.627	0.019	0.890	1.4	0.236	1.221	0.269	0.42	0.516	1.976	0.159	0.42	0.516	0.497	0.480	0.236	0.626	0.884	0.347	0.030	0.860

## Discussion

This is the first report of helminth and protozoan parasites found in the feces of *D. marsupialis* in the Corregimiento of Santa Elena, Medellín, Colombia. The study reported a wide diversity of gastrointestinal parasites in Colombia. The percentage of polyparasitism reported in this study was high because all individuals were co-infected with at least two parasites. This is consistent with the findings for *Didelphis virginiana* from Mexico (Aragón-Pech et al., 2018; Panti-May et al., 2024). Free-living animals are exposed to a wide diversity of parasites in their natural environment. In the face of these novel and increased risks of exposure to new parasites, improving our knowledge of the cumulative effects of co-infections and the role of polyparasitism in ecosystems is crucial (Bordes & Morand, 2009). Opossums are infected by at least 61 different helminth species over their geographical range (Alden, 1995); however, the question of whether parasites affect the health of opossums has not been fully investigated. In this study, signs compatible with severe parasitism, such as poor body condition, diarrhea, and malnutrition, reported in other studies (Jones, 2013; García-Valle et al., 2023) were absent.

The helminths with the highest prevalence in this study were *Cruzia* sp. Among *Cruzia* species, only *Cruzia tentaculata, C. cameroni*, and *C. americana* have been reported to parasitize marsupials of the Didelphidae subfamily (Souza et al., 2022). *Cruzia tentaculata* has been commonly reported as one of the most abundant species infecting *D. marsupialis* in South America (Jiménez et al., 2011; Chero et al., 2017); however, the health implications of this nematode in *D. marsupialis* are unknown. In Argentina, Santa-Cruz (2006) reported the presence of lesions associated with *C. tentaculata* in the cecum of *D. albiventris*, and Ramos-de-Souza et al. (2021) reported that a high infection rate of *C. americana,* which is closely related to *C. tentaculata*, may cause severe pathology or even death in *D. virginiana* in the United States. However, further studies are necessary to confirm this hypothesis.

Little is known about the life cycle of *Cruzia* spp. Recently, the giant African land snail (*Achatina fulica*), an invasive species, was found to be an intermediate host for *C. tentaculata* (Ramos-de-Souza et al., 2021). The generalist dietary habits of didelphid opossums and the presence of *A. fulica*, synonymous with *Lissachatina fulica*, in Antioquia (Penagos-Tabares et al., 2019), may favor the life cycle of *C. tentaculata*; hence, the eggs found in this study are probably *C. tentaculata* eggs. In Colombia, *C. tentaculata* specimens have been collected from the large intestine of *D. marsupialis* from Valle del Cauca, and Bucaramanga (Adnet et al., 2009; Betancourt-Echeverri et al., 2021), and this study would constitute the first record in Antioquia. Although we did not find significant statistical differences associated with host sex, we found that all females were infected with *C. tentaculata*. Similar results were found in *D. aurita* where a larger parasitic load of *C. tentaculata* in adult female hosts may be a consequence of the highly aggregated distribution of this parasite or females are most likely to accumulate parasites during their lifetime (Costa-Neto et al., 2019; Boullosa et al., 2021).

*Aspidodera* sp. was the second most prevalent helminth, which is in agreement with other studies on *D. marsupialis* (Jiménez et al., 2011; Freitas et al., 2022). Species of the genus *Aspidodera* are found in the cecum and large intestine of *Didelphis* spp. and are widely distributed throughout America (Chagas-Moutinho et al., 2014; Boullosa et al., 2021). Although *Aspidodera raillieti* has been widely reported in South America, including Colombia, little is known about its host-associated pathogenesis (Quintão e Silva & Costa, 1999; Chagas-Moutinho et al., 2007; Jiménez et al., 2011; Betancourt-Echeverri et al., 2021).

Several *Gnathostoma* species have been recorded from Mexico to Argentina; however, the only valid *Gnathostoma* species in opossums is *Gnathostoma turgidum* (Padilla Bejarano, 2021). This nematode was first described in Argentina in *D. albiventris* and subsequently mainly documented in *D. virginiana* from North America and *D. marsupialis* in Ecuador and Peru (Chero et al., 2017; García-Valle et al., 2023). *Gnathostoma turgidum* life cycle is heteroxenous and includes crustaceans, fish, and amphibians as intermediate hosts (Padilla Bejarano, 2021). Recently, possible zoonotic implications attributed to the accidental ingestion of third-stage larvae (L3) of *G. turgidum* were considered (Mosqueda-Cabrera et al., 2023). Clinical cases of cutaneous gnathostomiasis in Medellín residents, located close to the current study site, have been documented (Jurado et al., 2015). Therefore, molecular studies to identify the *Gnathostoma* species involved in this human infection are necessary.

*Turgida turgida* is a nematode that causes significant morbidity and mortality when present in large numbers in the stomach of opossums (Nichelason et al., 2008). We found a low prevalence when compared with other studies from Brazil in *D. albiventris* and *D. aurita* (Quintão e Silva & Costa, 1999; Costa-Neto et al., 2019); however, these data must be analyzed with caution since the previously mentioned results were obtained through necropsies of adult parasites. Interestingly, eggs of this parasite were found only in adult *D. marsupialis* females, but no statistically significant differences were found in relation to sex and age, which is consistent with other studies in which these parameters were evaluated (Costa-Neto et al., 2019; Boullosa et al., 2021; Freitas et al., 2022).

Helminth parasites of the order Trichinelida, with zoonotic potential, were detected in the opossums evaluated in this study. *Capillaria* and *Trichuris* have previously been reported in *D. albiventris, D. virginiana*, and *D. aurita* in Mexico and Brazil (Quintão e Silva & Costa, 1999; Aragón-Pech et al., 2018; Bezerra-Santos et al., 2020; Panti-May et al., 2024). Adult forms of *Capillaria* spp. and *Trichuris* spp. found in the large intestine of *D. marsupialis* have been described in the northeastern and southwestern regions of Colombia (Ramírez & Osorio, 2014; Betancourt-Echeverri et al., 2021). The *Strongyloides* sp. has been reported in *D. marsupialis* in Colombia (Ramírez & Osorio, 2014). In Neotropics, the advancement of agriculture and urbanization into more natural wild areas has increased human contact with wild animals or synanthropic species; consequently, the risk of infectious disease and zoonotic pathogen transmission has also increased (Jones & Tardieu, 2021).

In terms of marsupial cardiopulmonary nematodes, *Didelphostrongylus hayesi* and *Heterostrongylus heterostrongylus* have been reported to parasitize *D. marsupialis* and *D. aurita*, respectively (Costa-Neto et al., 2016). Pulmonary lesions have been associated with both types of lung worms (Costa-Neto et al., 2016; López-Crespo et al., 2017). Given the few morphological and molecular studies comparing the different species that parasitize the cardiopulmonary system of the opossum, we decided to classify the pulmonary larvae obtained in this study using the Baermann technique as belonging to the superfamily Metastrongyloidea; however, more studies are needed in this regard.

The Acanthocephalan helminth *O. microcephalus* exhibits a wide geographic distribution that extends from Brazil, where it was originally described, to as far north as the United States (Richardson et al., 2014). These endoparasites exhibit an indirect life cycle, in which possums, such as *D. marsupialis*, *D. virginiana,* and *Philander opossum*, are considered definitive hosts (Richardson, 2006; López-Caballero et al., 2015), and arthropods, such as millipedes, are intermediate hosts (Richardson, 2006). To the best of our knowledge, there is no previous record of didelphids naturally infected with *O. microcephalus* in Colombia, confirming that the *D. marsupialis* diet in the study area includes insects. We reported a low frequency with respect to the records reported from Argentina, Brazil, and Mexico, with prevalence ranges varying between 16.7–100% (Navone & Suriano, 1992; Aragón-Pech et al., 2018; Costa-Neto et al., 2019; Freitas et al., 2022; Panti-May et al., 2024).

In terms of protozoan parasites, *Eimeria* species have already been described within the family Didelphidae, including *Eimeria auritanenesis, E. didelphydis*, *E. gambai, E. indianenesis, E. marmosopos,* and *E. philanderi* (Valerio-Campos et al., 2015). Coccidia belonging to *Eimeria* were the most prevalent parasites in opossums in this study, as reported in previous studies (Aragón-Pech et al., 2018; Bezerra-Santos et al., 2020). However, despite the spread of this protozoan, how it affects host health is unclear (Duszynski, 2016). Sporulated oocysts have morphological and morphometric characteristics similar to *Eimeria marmorsopos* described in *D. marsupialis* from Central and South America (Heckscher et al., 1999; Valerio-Campos et al., 2015); however, we decided not to classify them within this genus because sporozoites and other internal structures could not be identified.

Considering the resistance of the oocyst or sporocyst wall, most Coccidia species can pass through the intestinal tract without any changes in morphological characteristics (Teixeira et al., 2003). The presence of *Octosporella* sp., which owes its name to having 8–12 sporocysts, is frequently observed in invertebrate hosts, and occasionally as a spurious parasite in the intestinal contents of vertebrate predators (Chinchilla-Carmona et al., 2013). We recorded for the first time the presence of this pseudoparasite in *D. marsupialis* feces, which indicates that these animals also include insects in their diets. Although more studies are necessary, the presence of Coccidia in didelphid feces appears to be frequent. In a survey of intestinal coccidiosis in 27 *D. aurita* adults from the southeastern region of Brazil, two individuals (7,41%) presented a large number of a polysporocystid oocysts (Teixeira et al., 2003). Another Brazilian study reported the prevalence of 1,8% (1/55) in *D. albiventris* (Teodoro et al., 2019).

In contrast, the presence of the flagellate protozoan *Giardia* sp. was found. Studies on this protozoan infecting didelphids are scarce, and most rely on identification at the genus level, rendering it difficult to assess their zoonotic potential (Bezerra-Santos et al., 2021). Studies by Zanette et al. (2008) on *D. albiventris* from Brazil demonstrated the presence of *Giardia* sp. cysts in 50% (3/6) of the samples. Other studies in the United States reported the presence of *Giardia* spp. in 14.7% of the *D. virginiana* opossums sampled using direct immunofluorescent antibody microscopy (Oates et al., 2012). In Colombia, Orozco-Cadenas (2018) found *Giardia* sp. in the fecal matter of a single *D. marsupialis* individual in captivity.

In the present study, five of the 10 helminth species collected from *D. marsupialis* had indirect patterns of transmission by intermediate host ingestion (Metastrongyloidea larvae, *C. tentaculata*, *G. turgidum*, *O. microcephalus*, and *T. turgida*), where arthropods and mollusks, which are present in the opossum diet, may act as intermediate hosts when infected with nematode larvae. Investigation of potential intermediate or paratenic hosts associated with the parasites found in *D. marsupialis* should be considered. Therefore, understanding the pathological effects of these parasites and the damage they cause to the host is necessary.

Overall, the results herein demonstrate for the first time the discovery of *Cruzia* sp., *Aspidodera* sp., *G. turgidum*, *Turgida* sp., *O. microcephalus*, and Metastrongyloidea eggs in a suburban area in Medellín, Colombia. Moreover, our study is the first report of pseudoparasitism by *Octosporella* spp. in *D. marsupialis*. Additionally, our study indicated that a high percentage of opossums were infected by potentially zoonotic parasites, such as Capillaridae nematodes, *Trichuris* spp., *Strongyloides* spp., and *Giardia* spp., implying that *D. marsupialis* may participate in zoonotic cycles in urban environments. Molecular studies that identify parasites at the species level should be considered in future. In addition, more frequent analyses of fecal material using the Baermann method could increase the database of helminths found in *D. marsupialis* in Colombia. Parasitological studies and their damage to the host are necessary to understand the ecological dynamics of host-parasite interactions and to develop programs for the conservation and protection of the species.
